# IL-27 induces autophagy through regulation of the DNMT1/lncRNA MEG3/ERK/p38 axis to reduce pulmonary fibrosis

**DOI:** 10.1186/s12931-023-02373-x

**Published:** 2023-03-03

**Authors:** Li Ting, Yingying Feng, Ying Zhou, Zhongkai Tong, Zhaoxing Dong

**Affiliations:** 1 Department of Respiratory and Critical Care Medicine, Ningbo Huamei Hospital, University of Chinese Academy of Sciences, Ningbo, Zhejiang China; 2 Department of Respiratory and Critical Care Medicine, Ningbo University, Ningbo, Zhejiang China

**Keywords:** IL-27, Autophagy, Pulmonary fibrosis, LncRNA MEG3, DNMT1

## Abstract

**Purpose:**

Previous studies have shown that interleukin-27 (IL-27) can reduce bleomycin (BLM)-induced pulmonary fibrosis (PF). However, the underlying mechanism by which IL-27 attenuates PF is not fully clear.

**Methods:**

In this research, we used BLM to construct a PF mouse model, and MRC-5 cells stimulated by transforming growth factor-β1 (TGF-β1) were used to construct a PF model in vitro. The lung tissue status was observed by Masson and hematoxylin and eosin (HE) staining. To detect gene expression, RT‒qPCR was used. The protein levels were detected by western blotting and immunofluorescence staining. EdU and ELISA were used to detect cell proliferation viability and hydroxyproline (HYP) content, respectively.

**Results:**

Aberrant IL-27 expression was observed in BLM-induced mouse lung tissues, and the use of IL-27 attenuated mouse lung tissue fibrosis. TGF-β1 induced autophagy inhibition in MRC-5 cells, and IL-27 alleviated MRC-5 cell fibrosis by activating autophagy. The mechanism is inhibition of DNA methyltransferase 1 (DNMT1)-mediated lncRNA MEG3 methylation and ERK/p38 signaling pathway activation. Overexpression of DNMT1, knockdown of lncRNA MEG3, autophagy inhibitor or ERK/p38 signaling pathway inhibitors reversed the positive effect of IL-27 in a lung fibrosis model in vitro.

**Conclusion:**

In conclusion, our study shows that IL-27 upregulates MEG3 expression through inhibition of DNMT1-mediated lncRNA MEG3 promoter methylation, which in turn inhibits ERK/p38 signaling pathway-induced autophagy and attenuates BLM-induced PF, providing a contribution to the elucidation of the potential mechanisms by which IL-27 attenuates PF.

**Supplementary Information:**

The online version contains supplementary material available at 10.1186/s12931-023-02373-x.

## Background

As a lung disease, pulmonary fibrosis (PF) is chronic, progressive and interstitial [1]. The main histopathological features of lung fibroblasts include fibroblasts losing control of proliferation and excessive accumulation of the extracellular matrix (ECM) deposited by muscle fibroblasts (differentiated from lung fibroblasts) [2]. Although the pathogenesis of PF has been extensively studied over the past decade, the exact cause of PF has not been elucidated. Our previous studies exploring the role of interleukin-27 (IL-27) in PF have demonstrated that IL-27 attenuates PF both in vivo and in vitro [3, 4], but the underlying mechanism by which IL-27 attenuates PF is unclear.

Autophagy is an intracellular degradation pathway that is essential for cell homeostasis and is evolutionarily conserved [5]. Some studies have shown that autophagy may be involved in PF diseases [6, 7]. In lung epithelial and lung fibroblast cells, reduced autophagic pathways were found in patients with idiopathic pulmonary fibrosis (IPF) [8]. In addition, autophagy is also involved in regulating the formation of ECM [9]. Del Principe et al. [10] found that autophagy deficiency promotes ECM deposition in lung fibroblasts and accelerates the fibrosis process. In addition, IL-27 can induce autophagy in macrophages [11]. However, it is not clear whether IL-27 can attenuate PF by inducing autophagy.

Increasing evidence shows that long noncoding RNAs (lncRNAs) have an important influence on PF [12, 13]. Gokey et al. [14] showed that in IPF epithelial cells, the most improved lncRNA is lncRNA maternally expressed gene 3 (MEG3), which plays an important role in regulating the function of basic progenitor cells. This may contribute to the organizational remodeling of IPF. In addition, Gao et al. [15] showed that MEG3 attenuates nickel oxide nanoparticle (NiO NP)-induced PF through regulation of Hedgehog signaling pathway-mediated autophagy. In eukaryotes, as a widely found family of serine/threonine protein kinases, MAPKs include p38, extracellular signal-regulated kinases (ERK), and c-Jun NH2-terminal kinase (JNK) [16]. ERK and p38 have also been shown to be involved in autophagy [17]. A recent study showed that the use of ERK and p38 pathway inhibitors significantly inhibited the expression of α-smooth muscle actin (α-SMA) and collagen production mediated by TGF-β1, thereby suppressing fibroblast differentiation and ECM production [18]. However, it is not known whether MEG3 induces autophagy by inhibiting the ERK/p38 pathway to attenuate PF.

Epigenetic changes affected by the environment and aging have an important effect on IPF [19, 20]. To date, changes, including DNA methylation, histone modification and noncoding RNA expression, are considered epigenetic modifications [21]. As the main epigenetic modification pathway in mammals, DNA methyltransferase 1 (DNMT1) is responsible for maintaining the methylation of related genes during DNA replication, thus affecting the expression of related genes [22]. DNMT1 is associated with the development of PF. DNMT1 is significantly highly expressed in silica and BLM-induced IPF, and inhibition of DNMT1 attenuates the extent of PF [23, 24]. In addition, DNMT1 inhibits MEG3 expression by mediating methylation of the MEG3 promoter region [25, 26]. However, whether DNMT1 affects the course of PF by influencing the promoter methylation of MGE3 is not known.

In this study, we sought to confirm that the attenuating effect of IL-27 on PF is produced through the induction of autophagy, which is regulated through the DNMT1/lncRNA MEG3/ERK/p38 axis. Our data suggest that hypomethylation of the MEG3 promoter can weaken PF because IL-27 can inhibit the methylation of the MEG3 gene mediated by DNMT1, which inhibits the ERK/p38 pathway to induce autophagy.

## Materials and methods

### BLM-induced PF

Six- to eight-week-old male C57BL/6 mice were obtained from the Animal Experiment Center of Kunming Medical University. Mice used for experiments were housed in a specific pathogen-free (SPF) environment. As previously mentioned [3], after a week of adaptive feeding, C57BL/6 mice were randomly divided into three groups: normal control group (given phosphate-buffered saline (PBS) buffer); BLM group (5 mg/kg BLM was dissolved in PBS and given to the mice through intratracheal instillation for a single time); IL-27 group (IL-27 recombinant protein was injected subcutaneously after BLM solution was given for a single time; 1 μg per mouse for 7, 14 and 28 days). All group mice were euthanized and sacrificed on days 7, 14 and 28 of treatment (five mice were sacrificed at each time period in each group). Lung tissues were collected for subsequent analysis. During the construction of the PF model, BLM administration caused death in mice, but the fatality rate was low, approximately 4%. All procedures of this study were performed according to the Helsinki Declaration of the World Medical Association, and the program was approved by the Ethics Committee of Kunming Medical University.

### Cell culture and treatment

The MRC-5 (human lung fibroblast-derived) cell line was purchased from the Chinese Academy of Sciences Cell Bank and cultured in minimum essential medium (MEM) containing 10% fetal bovine serum, penicillin (100 μg/mL) and streptomycin 100 (μg/mL). The cell lines were placed in a humidified atmosphere containing 5% CO_2_ at 37 °C. After pretreatment with PD98059 (1 μmol/L, TOCRIS, Bristol, UK), SB203580 (100 nmol/L, TOCRIS, Bristol, UK), or 3-methyladenine (20 mmol/L, 3-MA, Absin, Shanghai, China) for 2 h, MRC-5 cells were exposed to IL-27 (100 ng/mL, eBioscience, California, USA) and/or TGF-β1 (40 ng/mL, eBioscience, CA, USA) for 48 h.

### Cell transfection

In 6-well plates, cells were inoculated to 90% confluence (approximately 1 × 10^5^ cells/well) before transfection. Subsequently, 1 µg of plasmids (DNMT1, MEG3, sh-MEG3, sh-DNMT1 and the corresponding negative control plasmids, Sangon Biotech, Shanghai) were transfected into MRC-5 cells by Lipofectamine™ 2000 (Life Technologies, USA). Subsequently, the cells were placed at 37 °C and cultured under 5% CO_2_, and the transfection efficiency was measured for subsequent experiments.

### Tissue preparation and fibrosis assessment

After the mice were euthanized and sacrificed, part of the right lung tissues of mice were dissected and soaked in 4% paraformaldehyde for 2 days. Then, the sample was sliced into 5-µm-thick paraffin sections after dehydration and embedding. Subsequently, to evaluate alveolitis and PF, hematoxylin and eosin (HE) and Masson staining were used according to the methods in a previous report [27].

### Enzyme-linked immunosorbent assay (ELISA)

In this study, part of the right lung tissues of mice were taken using a tissue homogenizer, and PBS was added to 5 times the volume to fully grind on ice to collect tissue homogenates. The hydroxyproline (HYP) content was measured using an ELISA kit. The ELISA kit was purchased from CUSABIO and used according to the manufacturer's instructions.

### Methylation-specific PCR (MSP)

Consistent with the abovementioned method [26], the methylation level of the MEG3 gene promoter region was determined by a DNA methylation detection kit in this study. The PCR product was purified by a DNA purification kit, and the concentration of DNA was determined by a Nanodrop2000. The purified DNA was reacted with CT conversion reagent for desulfurization and purified again. Subsequently, the purified DNA was collected for MSP amplification. The procedure was as follows: predenaturation at 95 °C for 10 min, denaturation at 95 °C for 45 s, and methylation at 95 °C for 35 cycles. Finally, the PCR products were detected by agarose gel electrophoresis, stained with ethidium bromide and analyzed by an image analysis system.

### Chromatin immunoprecipitation

According to previous reports [26], 4% formaldehyde was used to fix cells, and the cells were disrupted by ultrasonication. After anti-DNMT1 antibody was added to the lysate, it was incubated for 2 h at 4 °C with rotation (100 rpm/min) to interact with the MEG3 promoter. Subsequently, 10 μL of Protein A agarose/SaLmon sperm DNA was added to the lysate and incubated for 2 h to precipitate the protein‒DNA. The beads were washed with NETN buffer three times to remove nonspecific binding. Subsequently, the complex rich in the MEG3 promoter was decrosslinked. Finally, the MEG3 promoter fragment was collected and purified for RT‒qPCR analysis.

### RT‒qPCR

In this study, part of mouse right lung tissue was homogenized, total RNA Extractor (Sangon Biotech) was used to extract total RNA from the lung tissues and MRC-5 cells, 1 μL of RNA samples was taken, RNA integrity detection was conducted on 1% agarose gel electrophoresis, and 1 μL of RNA samples was taken after dilution to measure the OD value through the ratio of OD260/OD280 to identify total RNA purity. A cDNA synthesis kit (Vazyme, Nanjing, China) was used to reverse transcribe 2 μg of mRNA into cDNA, and then the cDNA was used as the amplification template in a SYBR reaction system (Vazyme, Nanjing, China). Amplification procedure conditions: predenaturation at 94 °C for 2 min, 40 cycles at 94 °C for 15 s, and annealing at 60 °C for 30 s [28]. All primers used in this study were designed with Premier 5.0. The internal controls were U6 and GAPDH. The confidence of the PCR results was assessed by the dissociation curve and cycle threshold (CT) values. The results were calculated by the 2^−ΔΔCt^ method after being repeated at least 3 times.

### Ethynyl-2′-deoxyuridine (EdU) assay

In this study, an EdU proliferation test was used to evaluate the proliferation ability of MRC-5 cells by an EdU kit (RIBOBIO, Guangzhou, China). First, the EdU reagent was diluted according to the instructions, and then an appropriate amount was added to the cells and incubated for 2 h. The solution was discarded, PBS was used to wash the cells, and paraformaldehyde solution (4%) was used to fix the cells for 30 min. Then, the paraformaldehyde solution was discarded, and the cells were incubated with 2 mg/mL glycine solution on a decolorizing shaker for 5 min. Next, the fixative was poured off, and the cells were washed with PBS. The cells were then treated with 0.5% Triton X-100 and incubated on a shaker for 10 min. The cells were then washed with PBS once before adding previously prepared Apollo staining solution and destaining. The cells were incubated in the dark on a shaker for 30 min. The staining solution was discarded, the cells were rinsed with PBS, and DAPI was added for nuclear staining. Cells were then incubated in the dark for 30 min, and the staining solution was discarded. After washing with PBS, microscopic photography was performed under a fluorescence microscope.

### Western blot analysis

In this study, part of mouse right lung tissue was homogenized, the proteins from lung tissues and MRC-5 cells were extracted utilizing RIPA lysis buffer (Sangon Biotech, Shanghai), and a lysate containing phenylmethanesulfonyl fluoride (PMSF) was added. 50 μL of RIPA lysis and extraction buffer was added to the cultured cells and ground tissues, which were placed on ice for 20 min and centrifuged at 10,000 rpm for 10 min. The supernatant was transferred to a precooled EP tube, and a BCA assay (Sangon Biotech, Shanghai) was used to determine the total protein concentration. Proteins were denatured by 2 × SDS loading buffer, and the denatured protein was stored at − 80 °C. The target bands were transferred to polyvinylidene fluoride (PVDF) membranes by taking 50 μg for 10% SDS–polyacrylamide gel electrophoresis (SDS‒PAGE) and using skim milk powder (5%) to block the PVDF membrane for 2 h. PVDF membranes were incubated with the following Abcam antibodies for 12 h at 4 °C: p38 (1:1000), IL-27 (1:1000), α-SMA (1:5000), p-ERK (1:1000), fibronectin (FN; 1:500), collagen I (COL I; 1:5000), LC3B (1:1000), Beclin1 (1:2000), p-p38 (1:1000), collagen III (COL III; 1:1000), ERK (1:1000), and β-actin (1:5000). The secondary antibodies were added, and TBST buffer was used to wash the PVDF membranes. β-Actin was used as a control. Subsequently, chemiluminescent reagents were added, and the bands were analyzed for grayscale values using ImageJ software. Each experiment was repeated 3 times independently.

### Immunofluorescence assay

In this study, we used 24-well sterile slides to culture MRC-5 cells for 24 h. After allowing the cells to fuse to 60–70%, IL-27 (100 ng/mL) and/or TGF-β1 (40 ng/mL) were used to treat the cells for 48 h. Immunofluorescence assays were performed according to previous studies [29]. When incubation was complete, cells were washed three times with prechilled PBS before fixation with immunostaining fixative (Beyotime) for 30 min. Subsequently, cells were incubated with Triton X-100 (Beyotime) permeabilization buffer for 15 min and then blocked with QuickBlock™ (Beyotime) for 30 min at 37 °C. Then, primary antibodies against α-SMA, FN, COL1, LC3B and Beclin1 were incubated with the cells at 4 °C for 12 h. Subsequently, the appropriate fluorescein-conjugated secondary antibody was added to the cells and incubated. The cell nuclei were stained with DAPI. The samples were observed with a confocal microscope and photographed for analysis.

### Statistical analysis

GraphPad 8.0 was used to analyze and prepare graphs in this study. Experiments were set up with 3–5 samples/replicates per experiment/group/condition. Data are given as the mean ± SD. In statistical comparisons, Student’s t test was used when there were only two groups of differences. Moreover, one-way analysis of variance (ANOVA) followed by Tukey’s posttest for multiple comparisons was used to determine significant differences for groups of three or more. *P* values < 0.05 were considered statistically significant.

## Results

### IL-27 is aberrantly expressed in BLM-induced PF

On days 7, 14 and 28 of PF induction by BLM, mice were euthanized and sacrificed, and lung tissues were collected for analysis. The lung tissue in the BLM group was structurally disorganized based on HE and Masson staining. Pathological phenomena were observed, such as a thickened alveolar septum, infiltrating inflammatory cells, and a large number of collapsed alveoli, and the degree of PF became more severe with increasing induction time (Additional file 1: Fig. S1A). HYP can be used as an important indicator of collagen metabolism and the degree of interstitial fibrosis in the lung [30]. The results of the HYP assay showed a gradual increase in HYP content with increasing induction time compared to the NC group (Additional file 1: Fig. S1B). Collagen deposition is an important pathological feature of PF, especially COL I and COL III [31]. The expression levels of COL I and COL III gradually increased with increasing induction time, as shown by RT‒qPCR and Western blot results (Additional file 1: Figs. S1C–E). Similarly, the expression of IL-27 mRNA and protein significantly increased after day 7 of BLM induction but gradually decreased after days 14 and 28 of induction (Additional file 1: Fig. S1F, G). In conclusion, our findings suggest that BLM induction can induce PF in mice, and abnormal expression of IL-27 was observed at different induction times.

### IL-27 attenuates BLM-induced PF

Subsequently, we verified the effect of IL-27 on PF in vivo. Compared with the BLM group, HE staining results showed that alveolar septum thickness was reduced, alveolar inflammation was reduced and fibrosis was alleviated in the IL-27 group (Additional file 1: Fig. S2A). Masson staining results showed that IL-27 significantly reduced BLM-induced fibroblast activation and collagen matrix deposition (Additional file 1: Fig. S2B). The mRNA and protein expression levels of COL I and COL III were significantly reduced in the IL-27 group, as shown by RT‒qPCR and Western blot assays (Additional file 1: Fig. S2C–E).

### IL-27 attenuates TGF-β1-induced PF in vitro

**Fig. 1:**
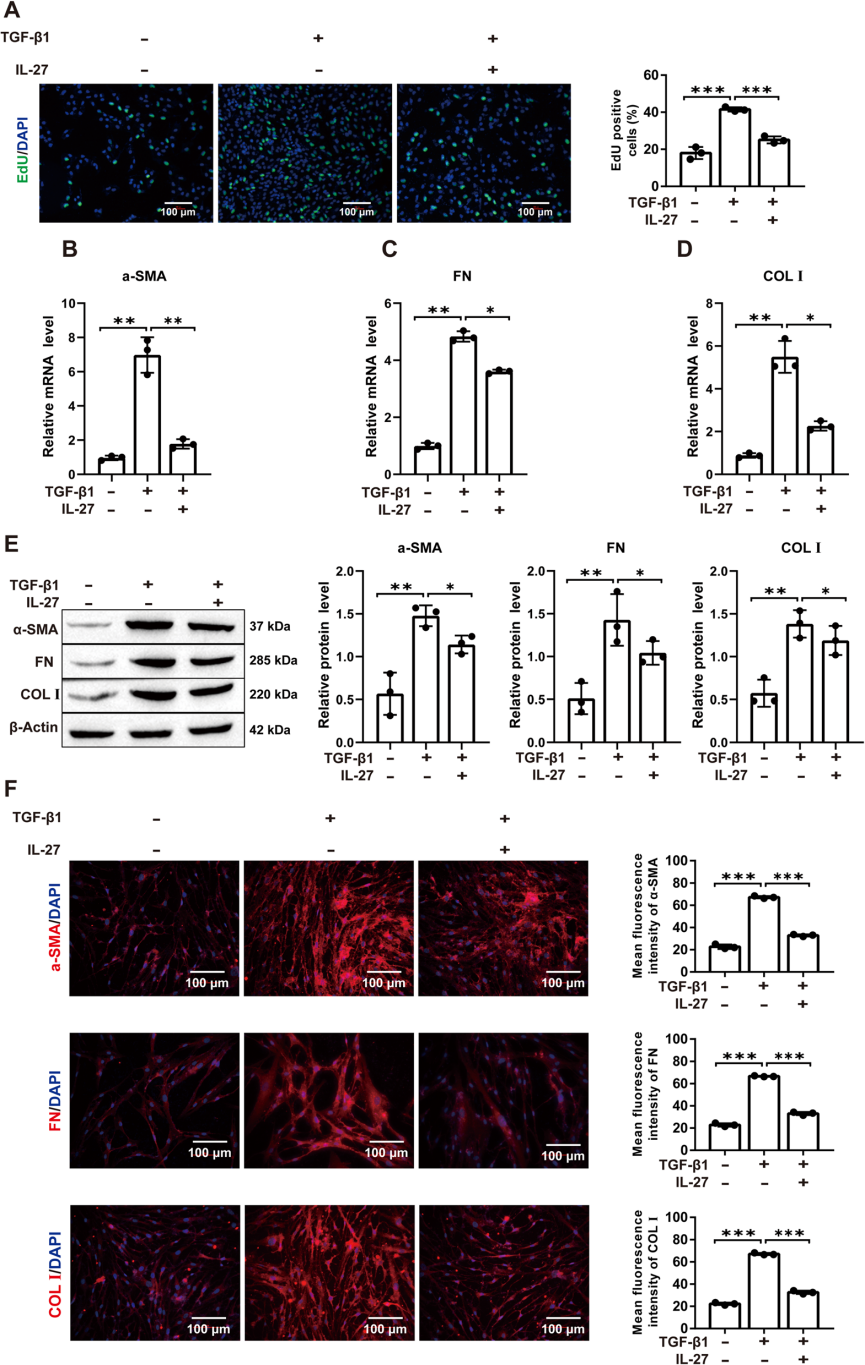
Effect of IL-27 on TGF-β1-induced fibrosis in MRC-5 cells. **A** EdU for cell proliferation viability (magnification: × 200; scale bars: 100 μm); **B**–**D** RT‒qPCR for the levels of fibrosis-related markers α-SMA, FN, COL I; **E** Western blot for the levels of α-SMA, FN, COL I; **F** Immunofluorescence detection of α-SMA, FN, and COL I fluorescence intensity (magnification: × 200; scale bars: 100 μm). *P < 0.05, **P < 0.01, ***P < 0.001; values are expressed as the mean ± standard deviation (n = 3)

First, we treated MRC-5 cells with TGF-β1 or IL-27 to examine the correlation between IL-27 and DNMT1, ERK/p38 signaling pathways and autophagy. RT‒qPCR assays showed that compared with untreated MRC-5 cells, IL-27 inhibited the expression level of DNMT1 (Additional file 1: Fig. S3A). Western blot assays showed that compared with untreated MRC-5 cells, IL-27 treatment inhibited the protein phosphorylation of ERK and p38 and increased the expression of the autophagy-related markers LC3 and Beclin1 (Additional file 1: Fig. S3B, C). These results suggest that IL-27 may affect the progression of PF by affecting DNMT1, ERK/p38 signaling pathways and autophagy. To further validate the role of IL-27 in PF, we used TGF-β1-induced MRC-5 cells to construct an in vitro PF model. The proliferation of lung fibroblasts and their differentiation into myofibroblasts are the main pathological changes in PF. α-SMA is a marker of myofibroblast activation [32], while FN is a component of the ECM [33]. Therefore, the levels of α-SMA and FN can respond to the degree of fibrosis. The proliferation viability of MRC-5 cells was significantly increased after TGF-β1 induction compared to the control group, but cell viability was significantly decreased again after treatment with IL-27 (Fig. 1A; Additional file 1: Fig. S4A). Compared to the control group, α-SMA, FN, and COL I mRNA and protein expression were significantly higher after TGF-β1 induction, but IL-27 treatment significantly decreased the levels of α-SMA, FN, and COL I (Fig. 1B–E). Similarly, immunofluorescence assays showed similar results, with significantly higher fluorescence intensity of α-SMA, FN, and COL I after TGF-β1 induction and lower fluorescence intensity after IL-27 treatment (Fig. 1F; Additional file 1: Fig. S4B). In conclusion, our results suggest that MRC-5 cell fibrosis is significantly increased after TGF-β1 induction and that IL-27 treatment attenuates TGF-β1-induced PF in vitro.

### IL-27 attenuates in vitro-induced PF through induction of autophagy

**Fig. 2:**
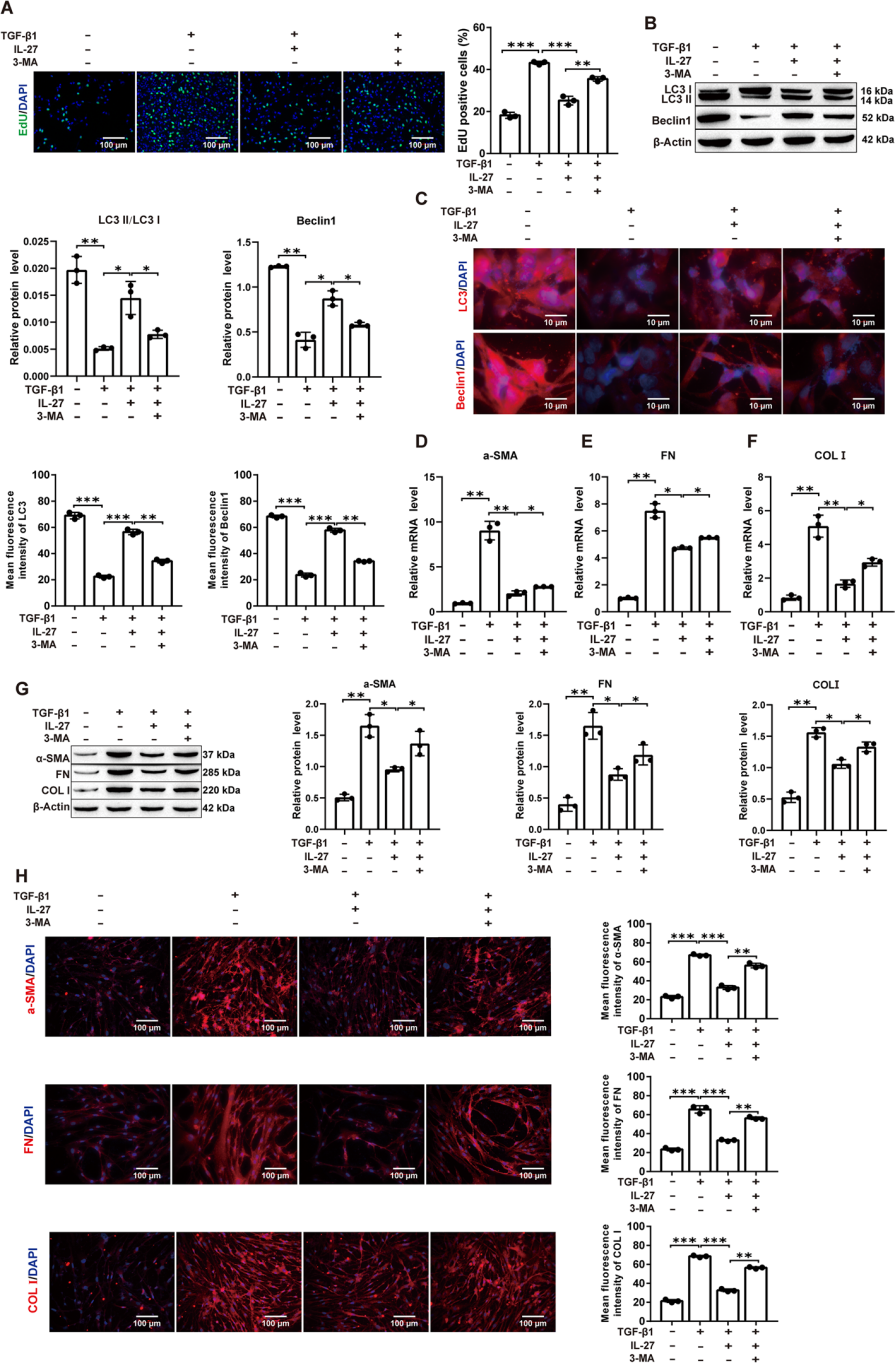
IL-27 attenuates TGF-β1-induced fibrosis in MRC-5 cells through activation of autophagy. **A** EdU for cell proliferation viability (magnification: × 200; scale bars: 100 μm); **B** Western blot detection of autophagy-related markers LC3 and Beclin1 protein expression levels; **C** Immunofluorescence detection of LC3 and Beclin1 fluorescence intensity (magnification: × 200; scale bars: 10 μm); **D**–**F** RT‒qPCR detection of α-SMA, FN, and COL I mRNA expression levels; **G** Western blot for α-SMA, FN, and COL I protein expression levels; **H** Immunofluorescence for α-SMA, FN, and COL I fluorescence intensity (magnification: × 200; scale bars: 100 μm). *P < 0.05, **P < 0.01, ***P < 0.001; values are expressed as the mean ± standard deviation (n = 3)

To verify whether IL-27 attenuates PF by inducing autophagy in MRC-5 cells, MRC-5 cells were cotreated with the autophagy inhibitor 3-MA and IL-27. EdU assay results showed that the inhibitory effect of IL-27 on cell proliferation was reversed by the addition of 3-MA (Fig. 2A; Additional file 1: Fig. S5A). Western blot assays of the autophagy-related markers LC3 and Beclin1 showed that protein expression was significantly reduced after TGF-β1 induction, which was significantly increased again after IL-27 treatment and significantly decreased again when IL-27 and 3-MA were cotreated (Fig. 2B). Similarly, the fluorescence intensity of LC3 and Beclin1 decreased after TGF-β1 induction and was reversed by IL-27. The fluorescence intensity of LC3 and Beclin1 was again significantly decreased when IL-27 and 3-MA were cotreated compared with the IL-27-treated group (Fig. 2C; Additional file 1: Fig. S5B). As expected, the mRNA and protein expression of the fibrosis-related proteins α-SMA, FN, and COL I significantly rebounded, and the fluorescence intensity was enhanced after 3-MA treatment compared with the IL-27 group (Fig. 2D–H; Additional file 1: Fig. S5C). These results showed that autophagy in MRC-5 cells was significantly reduced and fibrosis was elevated after TGF-β1 induction. In contrast, cellular autophagy levels were increased and fibrosis was decreased after IL-27 treatment. When 3-MA was used, it reversed IL-27-induced autophagy and increased the degree of fibrosis. In conclusion, IL-27 may attenuate PF induced in vitro by activating autophagy.

### IL-27-mediated inhibition of the ERK/p38 signaling pathway induces autophagy to attenuate PF induction in vitro

**Fig. 3:**
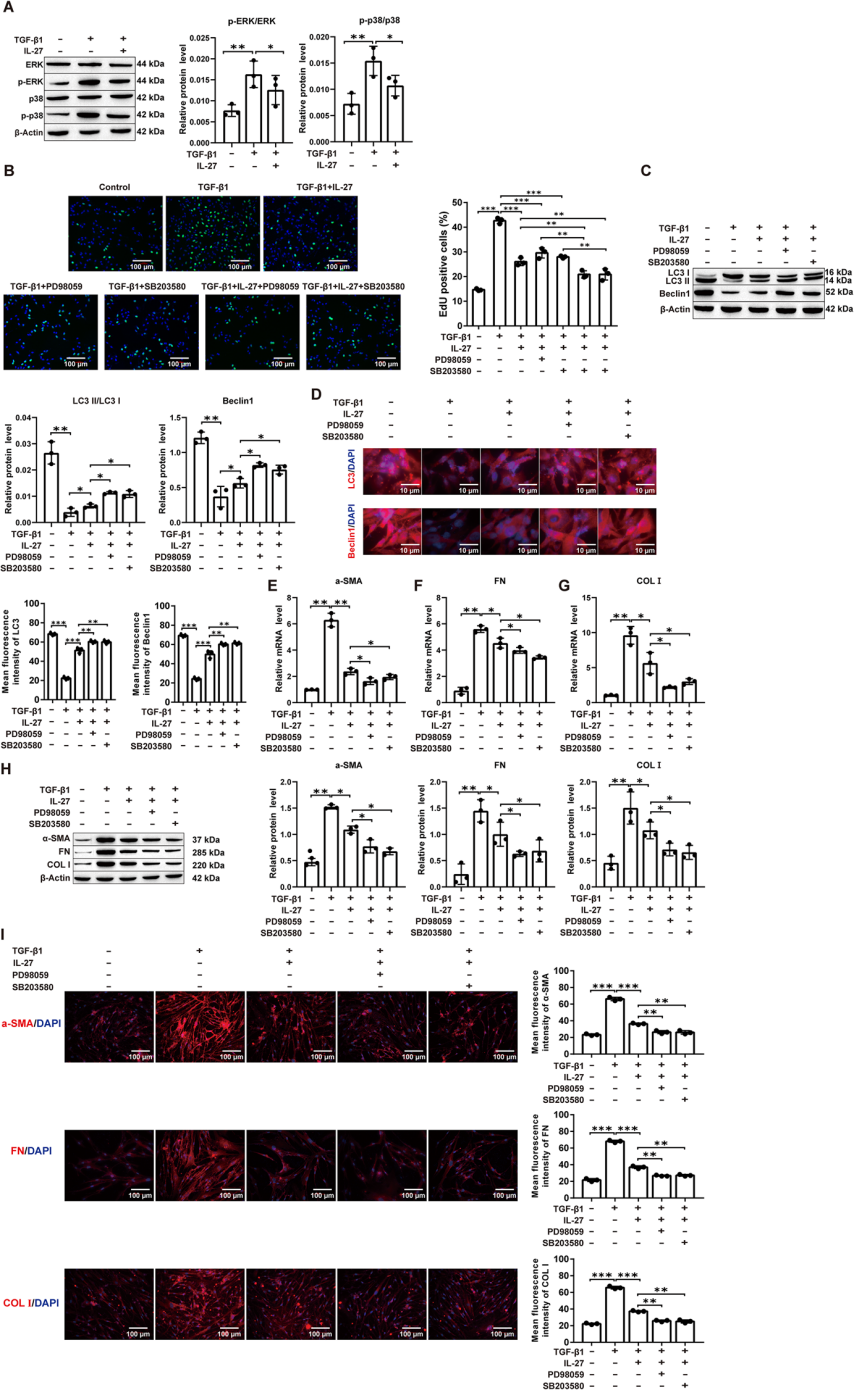
IL-27 attenuates TGF-β1-induced fibrosis in MRC-5 cells by activating autophagy through inhibition of the ERK/p38 signaling pathway. **A** Western blot for ERK and p38 phosphorylated protein levels; **B** EdU for cell proliferation viability (magnification: × 200; scale bars: 100 μm); **C** Detecting the level of autophagy-related markers LC3 and Beclin1 by Western blot; **D** Immunofluorescence for LC3 and Beclin1 fluorescence intensity (magnification: × 200; scale bars: 10 μm); **E**–**G** Detecting the mRNA levels of α-SMA, FN and COL I by RT‒qPCR; H: Western blot for α-SMA, FN and COL I protein levels; I: Immunofluorescence for α-SMA, FN and COL I fluorescence intensity (magnification: × 200; scale bars: 100 μm). *P < 0.05, **P < 0.01, ***P < 0.001; values are expressed as the mean ± standard deviation (n = 3)

To verify whether the ERK/p38 signaling pathway is involved in IL-27-induced autophagy, we treated TGF-β1-stimulated MRC-5 cells with IL-27 alone or together with the ERK inhibitor PD98059 or the p38 inhibitor SB203580. First, we observed that TGF-β1 treatment significantly elevated p-ERK/ERK and p-p38/p38 protein levels in MRC-5 cells and that IL-27 treatment reversed this increase (Fig. 3A). Proliferation assays revealed that treatment of MRC-5 cells with IL-27, ERK or p38 inhibitors prominently reduced cell proliferation induced by TGF-β1, and cotreatment with IL-27 and one of the inhibitors further decreased proliferation (Fig. 3B; Additional file 1: Fig. S6A). In line with this, Western blot and immunofluorescence analysis of autophagy markers revealed significant upregulation of LC3 and Beclin1 protein expression in IL-27 and ERK- or p38 inhibitor cotreated MRC-5 cells compared to IL-27 alone treated cells in response to TGF-β1 treatment (Fig. 3C, D; Additional file 1: Fig. S6B). RNA- and protein-level expression of ECM proteins such as α-SMA, FN, and COL I revealed a significant reduction upon IL-27 treatment, which was further lowered upon cotreatment with ERK or p38 inhibitors (Fig. 3E–I; Additional file 1: Fig. S6C). In addition, compared to ERK or p38 inhibitor treatment alone, cotreatment of IL-27 with one of the inhibitors significantly restored autophagy and inhibited profibrotic protein expression upon TGF-β1 treatment, thus further proving the additive effect of IL-27 and ERK or p38 inhibitors on these pathways (Additional file 1: Fig. S10A–E). In conclusion, these results confirm that IL-27 attenuates PF by activating autophagy via inhibition of the ERK/p38 signaling pathway.

### MEG3-mediated inhibition of the ERK/p38 signaling pathway induces autophagy to attenuate the induction of PF in vitro

**Fig. 4:**
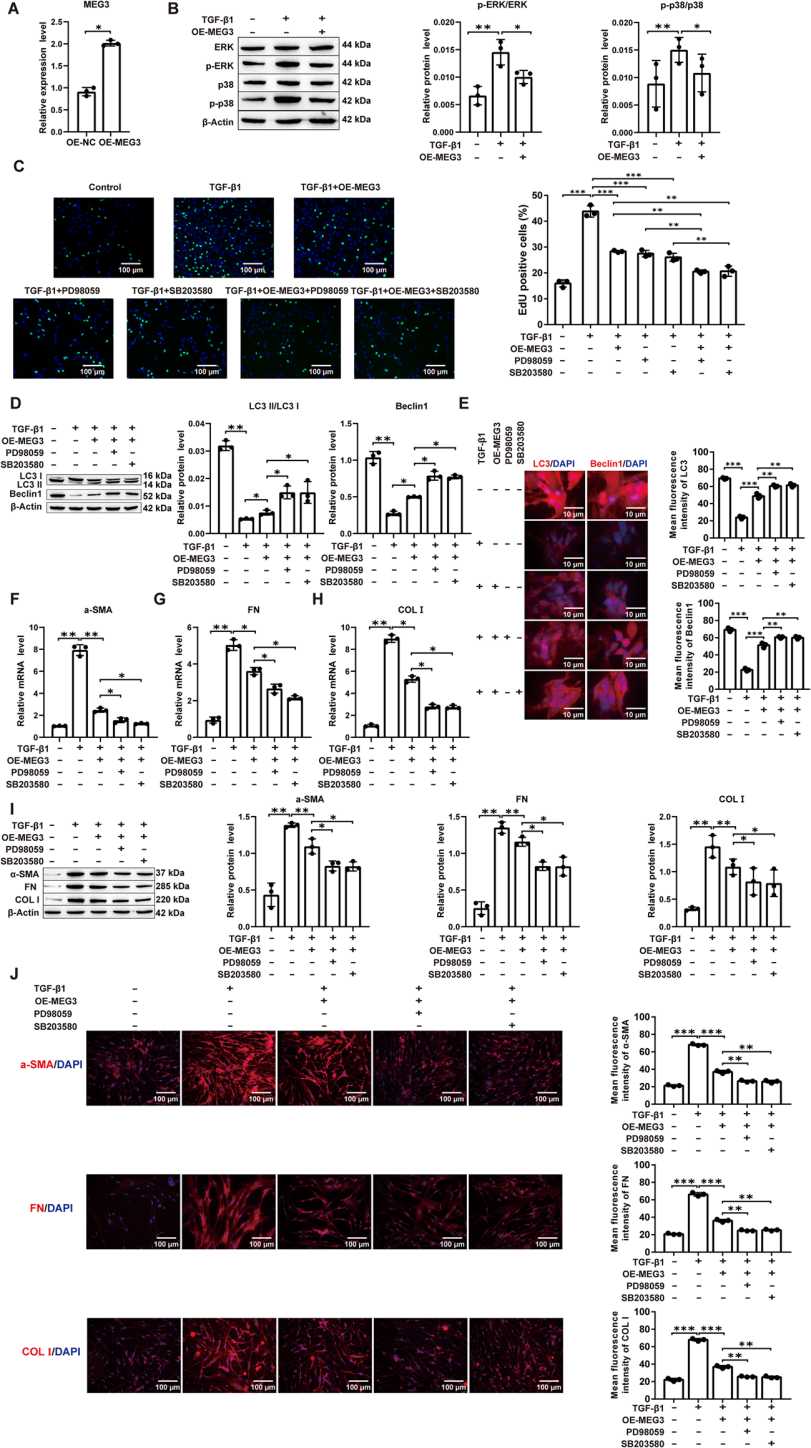
MEG3 attenuates TGF-β1-induced fibrosis by activating autophagy through inhibition of the ERK/p38 pathway in MRC-5 cells. **A** RT‒qPCR for MEG3 transfection efficiency; **B** Western blot for ERK and p38 phosphorylated protein expression levels; **C** EdU for cell proliferation viability (magnification: × 200; scale bars: 100 μm); **D** Western blot for LC3 and Beclin1 protein level; **E** Immunofluorescence detection of LC3 and Beclin1 fluorescence intensity (magnification: × 200; scale bars: 10 μm); **F**–**H** RT‒qPCR detection of α-SMA, FN, and COL I mRNA expression level; I: Western blot detection of α-SMA, FN, and COL I protein expression level; **J** Immunofluorescence detection of α-SMA, FN, and COL I fluorescence intensity (magnification: × 200; scale bars: 100 μm). *P < 0.05, **P < 0.01, ***P < 0.001; values are expressed as the mean ± standard deviation (n = 3)

Due to the aberrant expression of MEG3 in PF [14], we sought to verify whether MEG3 in MRC-5 attenuates PF by inhibiting the ERK/p38 signaling pathway to induce autophagy. First, the MEG3 plasmid and the corresponding negative control were transfected into MRC-5 cells. MEG3 expression levels were significantly increased in the MEG3 (OE-MEG3) group (Fig. 4A). TGF-β1 treatment significantly elevated p-ERK/ERK and p-p38/p38 protein levels in MRC-5 cells, and OE-MEG3 reversed this effect (Fig. 4B). Proliferation assays revealed that treatment of MRC-5 cells with OE-MEG3, ERK- or p38 inhibitor prominently reduced cell proliferation induced by TGF-β1, and cotreatment of OE-MEG3 and one of the inhibitors further decreased it (Fig. 4C; Additional file 1: Fig. S7A). Western blot and immunofluorescence analysis of autophagy markers revealed significant upregulation of LC3 and Beclin1 protein expression in OE-MEG3 and ERK- or p38 inhibitor cotreated MRC-5 cells compared to OE-MEG3 alone treated cells in response to TGF-β1 treatment (Fig. 4D, E; Additional file 1: Fig. S7B). In addition, RNA- and protein-level expression of ECM proteins such as α-SMA, FN, and COL I revealed a significant reduction upon OE-MEG3 treatment, which was further lowered upon cotreatment with ERK or p38 inhibitors (Fig. 4F–J; Additional file 1: Fig. S7C). In addition, compared to ERK or p38 inhibitor treatment alone, cotreatment of OE-MEG3 with one of the inhibitors significantly restored autophagy and inhibited profibrotic protein expression upon TGF-β1 treatment, thus further proving the additive effect of OE-MEG3 and ERK or p38 inhibitors on these pathways (Additional file 1: Fig. S11A–E). In conclusion, our results suggest that overexpression of MEG3 attenuates PF by activating autophagy via inhibition of the ERK/p38 signaling pathway in vitro.

### IL-27 inhibits DNMT1-mediated MEG3 methylation to upregulate MEG3 expression

**Fig. 5:**
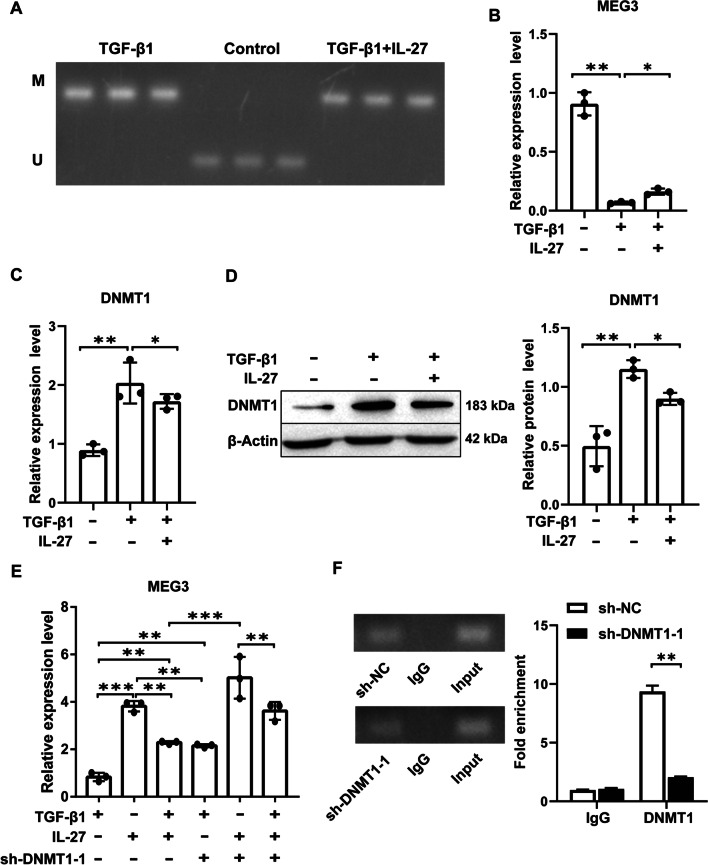
IL-27 upregulates MEG3 expression by inhibiting DNMT1-mediated methylation of the MEG3 promoter. **A **Detecting methylation in MRC-5 cells by MSP; **B** Detecting MEG3 mRNA levels in different treatment groups by RT‒qPCR; **C**, **D** Detecting DNMT1 expression in different treatment groups by RT‒qPCR and Western blotting; **E** RT‒qPCR to detect MEG3 expression levels; **F** chromatin immunoprecipitation to detect DNMT1 enrichment in the MEG3 promoter region. *P < 0.05, **P < 0.01, ***P < 0.001; values are expressed as the mean ± standard deviation (n = 3)

To verify whether MEG3 DNA methylation participates in the development of PF in TGF-β1-induced MRC-5 cells, we first examined whether DNA methylation occurred. The MSP assay showed a large amount of methylation after TGF-β1 treatment, while the degree of methylation decreased after treatment with IL-27 (Fig. 5A). Subsequently, MEG3 expression levels were detected using RT‒qPCR, and MEG3 expression was significantly reduced after TGF-β1 induction compared to the control, while IL-27 treatment reversed this process (Fig. 5B). In contrast, RT‒qPCR and Western blotting showed that DNMT1 expression was significantly elevated after TGF-β1 induction, while IL-27 treatment reversed the TGF-β1-induced DNMT1 elevation (Fig. 5C, D). Then, we examined whether DNMT1 mediates promoter methylation of MEG3. First, sh-NC, sh-DNMT1-1, sh-DNMT1-2 and sh-DNMT1-3 were transfected into TGF-β1-induced MRC-5 cells. sh-DNMT1-1 had the best influence on DNMT1 inhibition (Additional file 1: Fig. S8A, B). Therefore, sh-DNMT1-1 was used for subsequent experiments. In addition, RT‒qPCR results showed that compared with cells treated with TGF-β1, IL-27 cotreated with DNMT1 silencing significantly elevated MEG3 expression (Fig. 5E) and significantly reduced DNMT1 enrichment in the MEG3 promoter region (Fig. 5F). In conclusion, our results show that IL-27 can inhibit DNMT1-mediated methylation of the MEG3 promoter, thereby upregulating MEG3 expression.

### DNMT1 overexpression or MEG3 silencing reverses IL-27-induced autophagy to attenuate the protective effect of PF

**Fig. 6:**
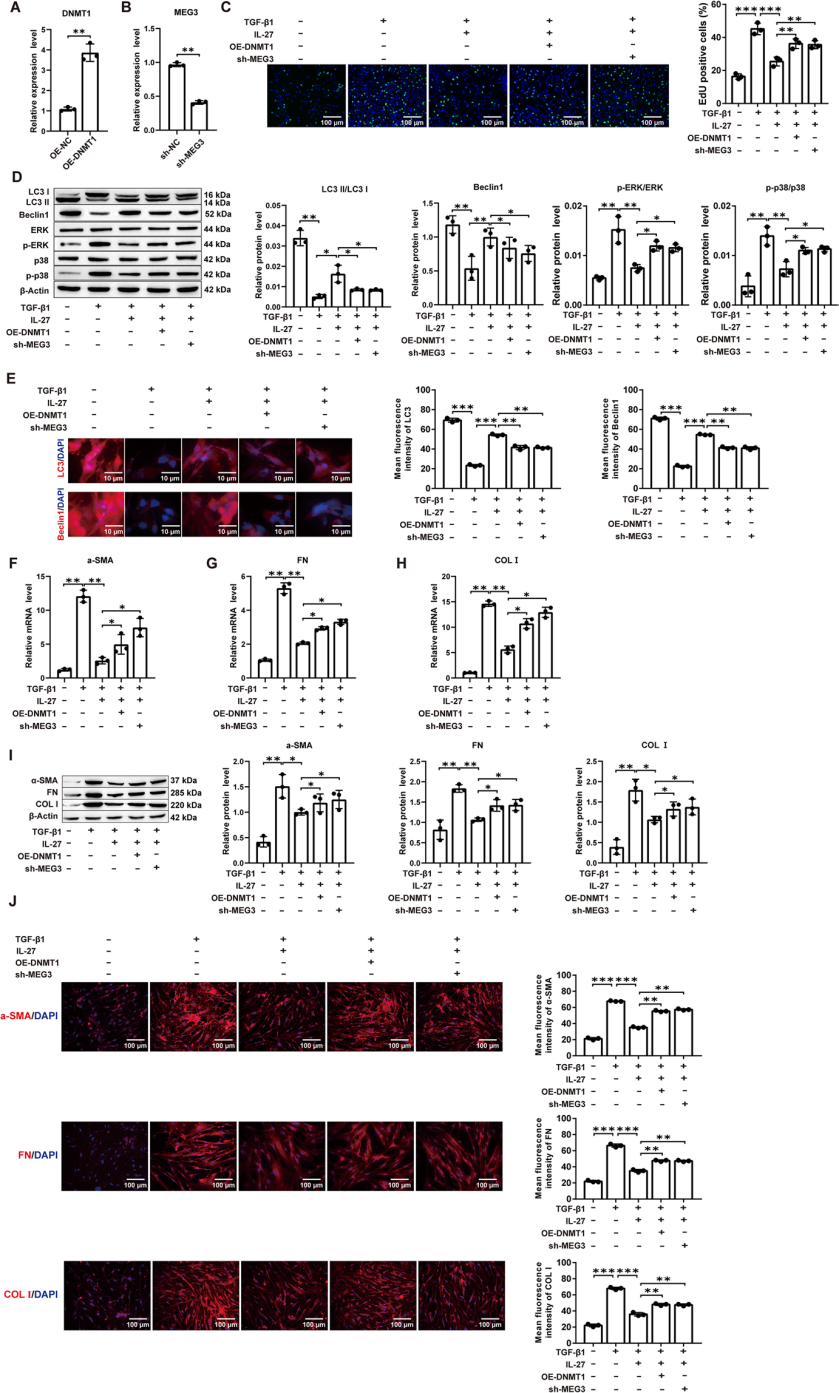
DNMT1 overexpression or MEG3 silencing reversed IL-27-activated autophagy to attenuate TGF-β1-induced fibrosis in MRC-5 cells. **A**, **B** RT‒qPCR for DNMT1 and MEG3 transfection efficiency; **C** EdU for cell proliferation viability (magnification: × 200; scale bars: 100 μm); **D** Western blot for protein expression levels; **E** Immunofluorescence for LC3 and Beclin1 fluorescence intensity (magnification: × 200; scale bars: 10 μm); **F**–**H** The mRNA levels of α-SMA, FN, and COL I were detected by RT‒qPCR; **I** Western blot to detect α-SMA, FN, and COL I protein expression levels; **J** Immunofluorescence to detect α-SMA, FN, and COL I fluorescence intensity (magnification: × 200; scale bars: 100 μm). *P < 0.05, **P < 0.01, ***P < 0.001; values are expressed as the mean ± standard deviation (n = 3)

In this study, DNMT1 overexpression (OE-DNMT1) and MEG3 silencing (sh-MEG3) plasmids and negative controls (OE-NC and sh-NC) were transfected into MRC-5 cells. DNMT1 expression was significantly increased in the OE-DNMT1 group compared to the OE-NC group (Fig. 6A). Compared to the sh-NC group, the sh-MEG3 group significantly decreased the expression of MEG3 (Fig. 6B). EdU test results showed that the inhibition of IL-27 on cell viability was reversed by adding OE-DNMT1 or sh-MEG3 (Fig. 6C; Additional file 1: Fig. S9A). Subsequently, Western blot assays showed that either DNMT1 overexpression or MEG3 silencing reversed the promotion of LC3 and Beclin1 protein expression and the inhibition of p-ERK and p-38 protein expression by IL-27 compared with the IL-27 group (Fig. 6D). The immunofluorescence assays showed that either DNMT1 overexpression or MEG3 silencing reversed the promotion of LC3 and Beclin1 fluorescence intensity by IL-27 compared with the IL-27 group (Fig. 6E; Additional file 1: Fig. S9B). In addition, RT‒qPCR, Western blot and immunofluorescence detection of α-SMA, FN, and COL I expression showed that DNMT1 overexpression or MEG3 silencing reversed the promotion of IL-27 on α-SMA, FN, and COL I mRNA and protein expression as well as fluorescence intensity compared with the IL-27 group (Fig. 6F–J; Additional file 1: Fig. S9C). In conclusion, our results show that either DNMT1 overexpression or MEG3 silencing reversed the protective effect of IL-27-induced autophagy to attenuate PF.

## Discussion

Consistent with previous findings [3], our results showed that the level of IL-27 in BLM-induced PF mice first increased and then gradually decreased. The attenuating effect of IL-27 on PF may be produced by inhibiting DNMT1-mediated methylation of the MEG3 promoter region, thereby upregulating MEG3 expression and inhibiting the ERK/p38 signaling pathway to induce autophagy.

Autophagy is a self-degrading process [34]. During PF, overactivation of lung fibroblasts becomes an important pathogenic process [35]. Regardless of the type of fibrosis, including PF, hepatic fibrosis and renal fibrosis, excessive deposition of ECM is a key disease feature [36–38]. The excessive activation of pulmonary fibroblasts synthesizes ECM components, especially collagen, which in turn promotes fibrosis [39]. In addition, impaired autophagic flux has been observed in TGF-β1-stimulated lung fibroblasts [40]. More importantly, a necessary and sufficient condition for the maintenance of normal lung fibroblast fate is the promotion of autophagy [41]. Therefore, the induction of autophagy in lung fibroblasts could somehow attenuate the course of PF. In this study, in TGF-β1-induced MRC-5 cells, the autophagy-related marker proteins LC3 and Beclin1 were significantly reduced, and IL-27 treatment-induced cellular autophagy and the proliferation viability of MRC-5 cells were inhibited. IL-27-induced cellular autophagy and protection against PF were reversed when the autophagy inhibitor 3-MA was used. In addition, as a multieffect cytokine, IL-27 is related not only to autophagy but also to oxidative stress and inflammatory reactions and plays a corresponding role in many diseases [42, 43]. Both inflammation and oxidative stress are crucial in inducing the progression of PF, and inhibiting inflammation and oxidative stress can reduce BLM-induced PF [44, 45]. In this study, combined with the previous discussion, we found that IL-27 can promote autophagy in MRC-5 cells and reduce the expression of TGF-β1-induced fibrosis markers. This expression was partially restored by the addition of 3-MA. However, whether IL-27 participates in inflammatory reactions, oxidative stress or other possible mechanisms is unknown and is worthy of further exploration in future experiments.

The MAPK signaling pathway plays a key role in fibrosis in many major organs, such as myocardial fibrosis, renal fibrosis, and PF [46]. Epithelial mesenchymal transition (EMT) is a key step in fibrosis [47]. The MAPK signaling pathway has been found to mediate paraquat-induced EMT in alveolar epithelial cells to promote PF [48]. In addition, Wang et al. [49] showed that the phosphorylation levels of JNK, p38 and ERK were significantly elevated in both BLM-induced PF and TGF-β1-stimulated MRC-5 cells and that the MAPK signaling pathway plays a key role in the inhibition of PF. Similarly, Li et al. [50] showed that p38 phosphorylation was significantly increased in TGF-β1-stimulated human embryonic lung fibroblasts (HLFs) and that the use of p38 MAPK inhibitors inhibited TGF-β1-stimulated HLF proliferation, induced HLF autophagy, and attenuated PF [51]. In MRC-5 cells, we found that the levels of ERK and p38 induced by TGF-β1-induced phosphorylation were increased, and IL-27 treatment reversed this process. Meanwhile, the use of ERK and p38 inhibitors further promoted the protective effect of IL-27 on PF.

DNA methylation is associated with the pathogenesis and progression of PF [52]. The prevalence of DNA methylation in PF has been confirmed by DNA methylation analysis and targeted gene studies [53]. DNMT1 is one of the most important DNA methylation transferases [54]. It can influence the growth of fibrosis by influencing the hypermethylation of specific genes [55]. Studies declared that the downregulated MEG3 could be an important indicator of fibrotic diseases, and further mechanism analysis revealed that the overexpressed MEG3 alleviated the progression of distinct organ fibrosis [56]. DNMT1 regulates MEG3 expression by altering the methylation level of the MEG3 promoter in TGFβ1-induced renal fibrosis, thereby affecting the development of renal fibrosis [57]. In addition, Gao et al. [15] showed that MEG3 attenuates nickel oxide nanoparticle (NiO NP)-induced PF through regulation of Hedgehog signaling pathway-mediated autophagy. However, whether DNMT1 mediates MEG3 promoter hypermethylation to affect the development of PF is unclear. In TGF-β1-induced MRC-5 cells, DNA hypermethylation occurred, and DNMT1 expression was upregulated and MEG3 was downregulated after TGF-β1 stimulation, a result that was reversed by treatment with IL-27. Furthermore, we demonstrated that DNMT1 affects MEG3 expression by mediating MEG3 promoter hypermethylation, and IL-27 inhibits DNMT1-mediated MEG3 promoter hypermethylation, thereby upregulating MEG3 expression and thus inhibiting the ERK/p38 pathway to attenuate TGF-β1-induced PF in vitro.

In conclusion, our study shows that IL-27 upregulates MEG3 expression by inhibiting DNMT1-mediated lncRNA MEG3 promoter methylation, which in turn inhibits the ERK/p38 signaling pathway to induce autophagy to attenuate BLM-induced PF, that provides help to elucidate the potential mechanism by which IL-27 attenuates PF.

## Supplementary Information

**Additional file 1: Figure S1** BLM induced PF in mice, and abnormal IL-27 expression was observed. A: HE and Masson staining to detect histological changes in the lung after 7, 14 and 28 days of BLM induction (magnification: × 200; scale bars: 100 μm); B: ELISA to detect hydroxyproline content; C, D and E: COL I and III expression in tissues by RT‒qPCR and Western blot; F and G: Detecting IL-27 expression in tissues by RT‒qPCR and Western blot. *P < 0.05, **P < 0.01, ***P < 0.001; values are expressed as the mean ± standard deviation (n = 5). **Figure S2** IL-27 attenuates BLM-induced PF in mice. A and B: HE and Masson staining to detect histological changes in the lungs of different treatment groups after induction for 7, 14 and 28 days (magnification: × 200; scale bars: 100 μm); C, D and E: Detection of the expression of COL I and III in different treatment groups by RT‒qPCR and Western blotting. *P < 0.05, **P < 0.01, ***P < 0.001; values are expressed as the mean ± standard deviation (n = 5). **Figure S3** Effects of IL-27 on DNMT1, ERK/p38 signaling pathways and autophagy. A: The mRNA levels of DNMT1 were detected by RT‒qPCR; B: Western blot for ERK and p38 phosphorylated protein levels; C: Western blot for LC3 and Beclin1 protein levels. *P < 0.05, **P < 0.01, ***P < 0.001; values are expressed as the mean ± standard deviation (n = 3). **Figure S4**. EdU for cell proliferation viability and immunofluorescence to detect α-SMA, FN, and COL I fluorescence intensity. A: EdU for cell proliferation viability (magnification: × 200; scale bars: 100 μm); B: Immunofluorescence to detect α-SMA, FN, and COL I fluorescence intensity (magnification: × 200; scale bars: 100 μm). **Figure S5**. EdU for cell proliferation viability and immunofluorescence to detect LC3, Beclin1, α-SMA, FN, and COL I fluorescence intensity. A: EdU for cell proliferation viability (magnification: × 200; scale bars: 100 μm); B: Immunofluorescence to detect LC3 and Beclin1 fluorescence intensity (magnification: × 200; scale bars: 10 μm); C: Immunofluorescence to detect α-SMA, FN, and COL I fluorescence intensity (magnification: × 200; scale bars: 100 μm). **Figure S6** EdU for cell proliferation viability and immunofluorescence to detect LC3, Beclin1, α-SMA, FN, and COL I fluorescence intensity. A: EdU for cell proliferation viability (magnification: × 200; scale bars: 100 μm); B: Immunofluorescence to detect LC3 and Beclin1 fluorescence intensity (magnification: × 200; scale bars: 10 μm); C: Immunofluorescence to detect α-SMA, FN, and COL I fluorescence intensity (magnification: × 200; scale bars: 100 μm). **Figure S7** EdU for cell proliferation viability and immunofluorescence to detect LC3, Beclin1, α-SMA, FN, and COL I fluorescence intensity. A: EdU for cell proliferation viability (magnification: × 200; scale bars: 100 μm); B: Immunofluorescence to detect LC3 and Beclin1 fluorescence intensity (magnification: × 200; scale bars: 10 μm); C: Immunofluorescence to detect α-SMA, FN, and COL I fluorescence intensity (magnification: × 200; scale bars: 100 μm). **Figure S8** Detecting DNMT1 expression after sh-DNMT1 1–3 treatment by RT‒qPCR and Western blotting. A: Detecting DNMT1 expression after sh-DNMT1 1–3 treatment by RT‒qPCR; B: Detecting DNMT1 expression after sh-DNMT1 1–3 treatment by Western blotting. **Figure S9** EdU for cell proliferation viability and immunofluorescence to detect LC3, Beclin1, α-SMA, FN, and COL I fluorescence intensity. A: EdU for cell proliferation viability (magnification: × 200; scale bars: 100 μm); B: Immunofluorescence to detect LC3 and Beclin1 fluorescence intensity (magnification: × 200; scale bars: 10 μm); C: Immunofluorescence to detect α-SMA, FN, and COL I fluorescence intensity (magnification: × 200; scale bars: 100 μm). **Figure S10** Detecting LC3, Beclin1, α-SMA, FN, and COL I by RT‒qPCR and Western blotting. A: Western blot for LC3 and Beclin1 protein levels; B, C and D: RT‒qPCR detection of α-SMA, FN, and COL I mRNA expression levels; E: Western blot detection of α-SMA, FN, and COL I protein expression levels. *P < 0.05, **P < 0.01, ***P < 0.001; values are expressed as the mean ± standard deviation (n = 3). **Figure S11** Detecting LC3, Beclin1, α-SMA, FN, and COL I by RT‒qPCR and Western blotting. A: Western blot for LC3 and Beclin1 protein levels; B, C and D: RT‒qPCR detection of α-SMA, FN, and COL I mRNA expression levels; E: Western blot detection of α-SMA, FN, and COL I protein expression levels. *P < 0.05, **P < 0.01, ***P < 0.001; values are expressed as the mean ± standard deviation (n = 3).

## Data Availability

The datasets used and/or analyzed during the current study are available from the corresponding author upon reasonable request.

## Abbreviations

IL-27: Interleukin-27
BLM: Bleomycin
PF: Pulmonary fibrosis
TGF-β1: Transforming growth factor-β1
HE: Hematoxylin and eosin
HYP: Hydroxyproline
DNMT1: DNA methyltransferase 1
ECM: Extracellular matrix
IPF: Idiopathic pulmonary fibrosis
lncRNAs: Long noncoding RNAs
MEG3: LncRNA MEG3
NiO NPs: Nickel oxide nanoparticles
ERK: Extracellular signal-regulated kinases
JNK: c-Jun NH2-terminal kinase
α-SMA: α-smooth muscle actin
SPF: Specific pathogen-free
PBS: Phosphate-buffered saline
MEM: Minimum essential medium
3-MA: 3-Methyladenine
MSP: Methylation-specific
PCR CT: Cycle threshold
PMSF: Phenylmethanesulfonyl fluoride
PVDF: Polyvinylidene fluoride
SDS‒PAGE: SDS-polyacrylamide gel electrophoresis
FN: Fibronectin
COL I: Collagen I
COL III: Collagen III
EMT: Epithelial mesenchymal transition
HLFs: Human embryonic lung fibroblasts
KLF4: Krüppel-like factor 4
