# Supplementary material for: IL-27 induces autophagy through regulation of the DNMT1/lncRNA MEG3/ERK/p38 axis to reduce pulmonary fibrosis
Source: Respir Res. 2023 Mar 3;24:67. doi: 10.1186/s12931-023-02373-x (PMC9985266; doi:10.1186/s12931-023-02373-x)
Supplement: Supplementary file 1 — Additional file 1: Figure S1 BLM induced PF in mice, and abnormal IL-27 expression was observed. A: HE and Masson staining to detect histological changes in the lung after 7, 14 and 28 days of BLM induction (magnification: × 200; scale bars: 100 μm); B: ELISA to detect hydroxyproline content; C, D and E: COL I and III expression in tissues by RT‒qPCR and Western blot; F and G: Detecting IL-27 expression in tissues by RT‒qPCR and Western blot. *P < 0.05, **P < 0.01, ***P < 0.001; values are expressed as the mean ± standard deviation (n = 5). Figure S2 IL-27 attenuates BLM-induced PF in mice. A and B: HE and Masson staining to detect histological changes in the lungs of different treatment groups after induction for 7, 14 and 28 days (magnification: × 200; scale bars: 100 μm); C, D and E: Detection of the expression of COL I and III in different treatment groups by RT‒qPCR and Western blotting. *P < 0.05, **P < 0.01, ***P < 0.001; values are expressed as the mean ± standard deviation (n = 5). Figure S3 Effects of IL-27 on DNMT1, ERK/p38 signaling pathways and autophagy. A: The mRNA levels of DNMT1 were detected by RT‒qPCR; B: Western blot for ERK and p38 phosphorylated protein levels; C: Western blot for LC3 and Beclin1 protein levels. *P < 0.05, **P < 0.01, ***P < 0.001; values are expressed as the mean ± standard deviation (n = 3). Figure S4. EdU for cell proliferation viability and immunofluorescence to detect α-SMA, FN, and COL I fluorescence intensity. A: EdU for cell proliferation viability (magnification: × 200; scale bars: 100 μm); B: Immunofluorescence to detect α-SMA, FN, and COL I fluorescence intensity (magnification: × 200; scale bars: 100 μm). Figure S5. EdU for cell proliferation viability and immunofluorescence to detect LC3, Beclin1, α-SMA, FN, and COL I fluorescence intensity. A: EdU for cell proliferation viability (magnification: × 200; scale bars: 100 μm); B: Immunofluorescence to detect LC3 and Beclin1 fluorescence intensity (magnification: × 200 [file 12931_2023_2373_MOESM1_ESM.docx]

Additional file 1


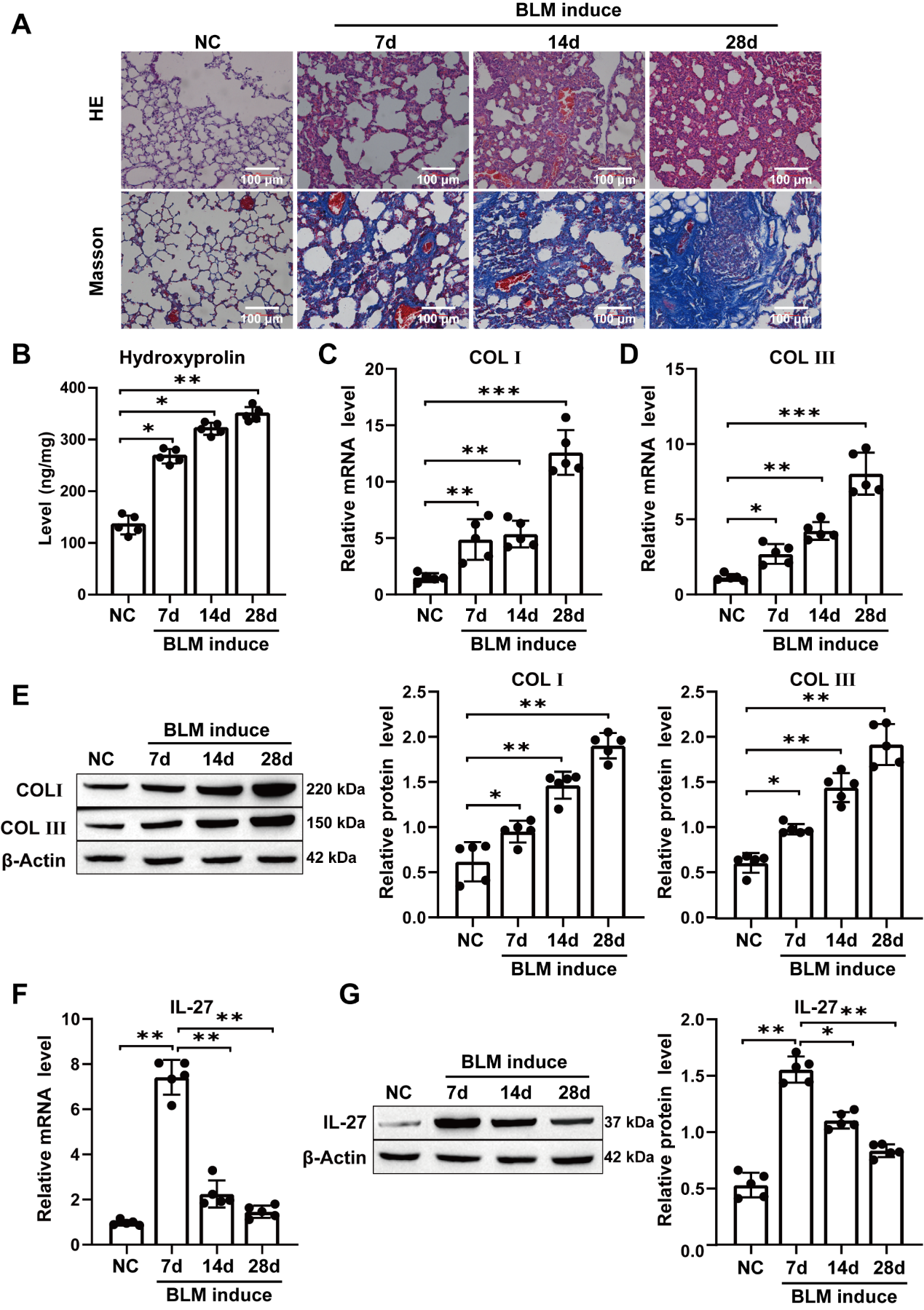


Figure S1 BLM induced PF in mice, and abnormal IL-27 expression was observed


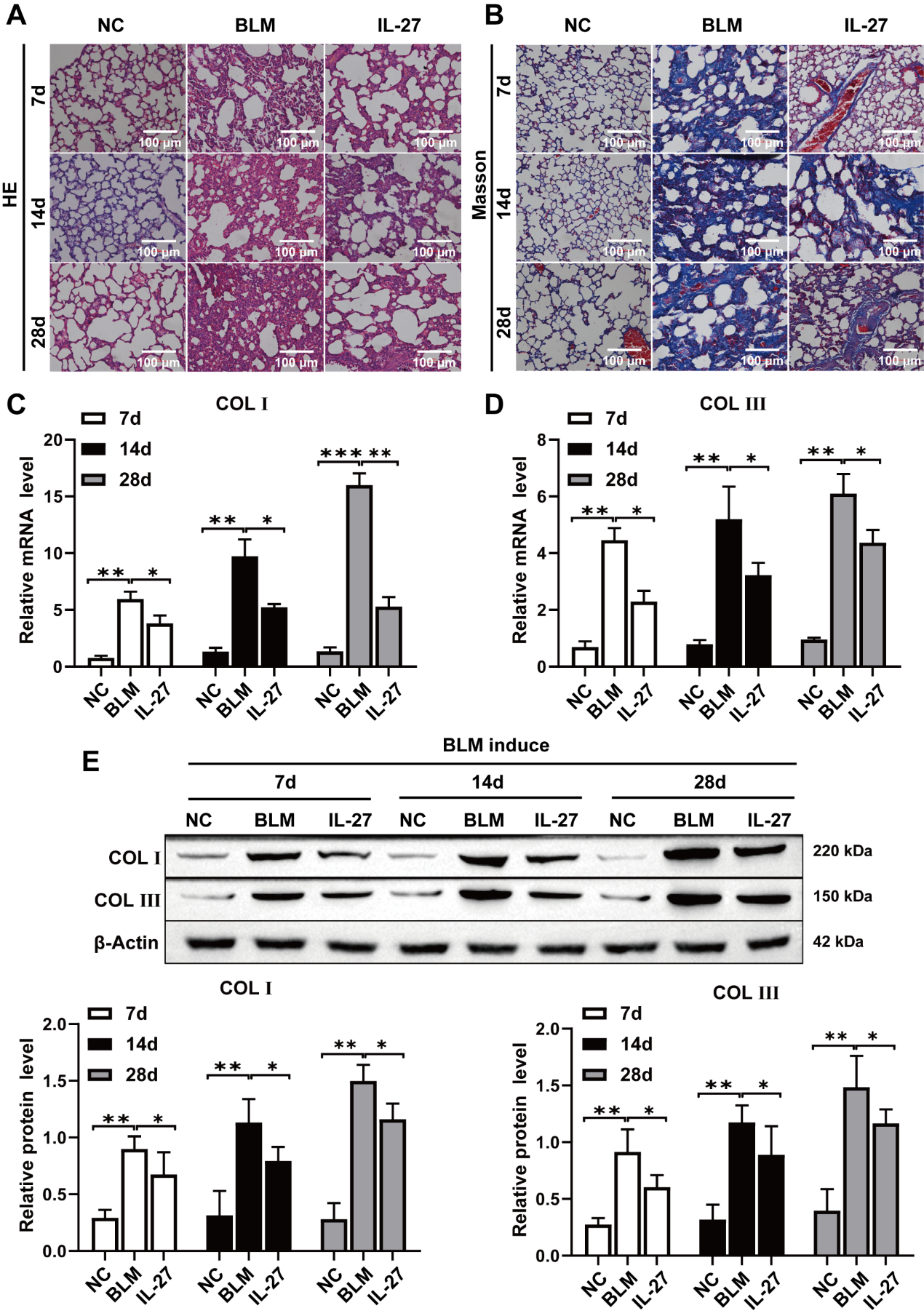


Figure S2 IL-27 attenuates BLM-induced PF in mice


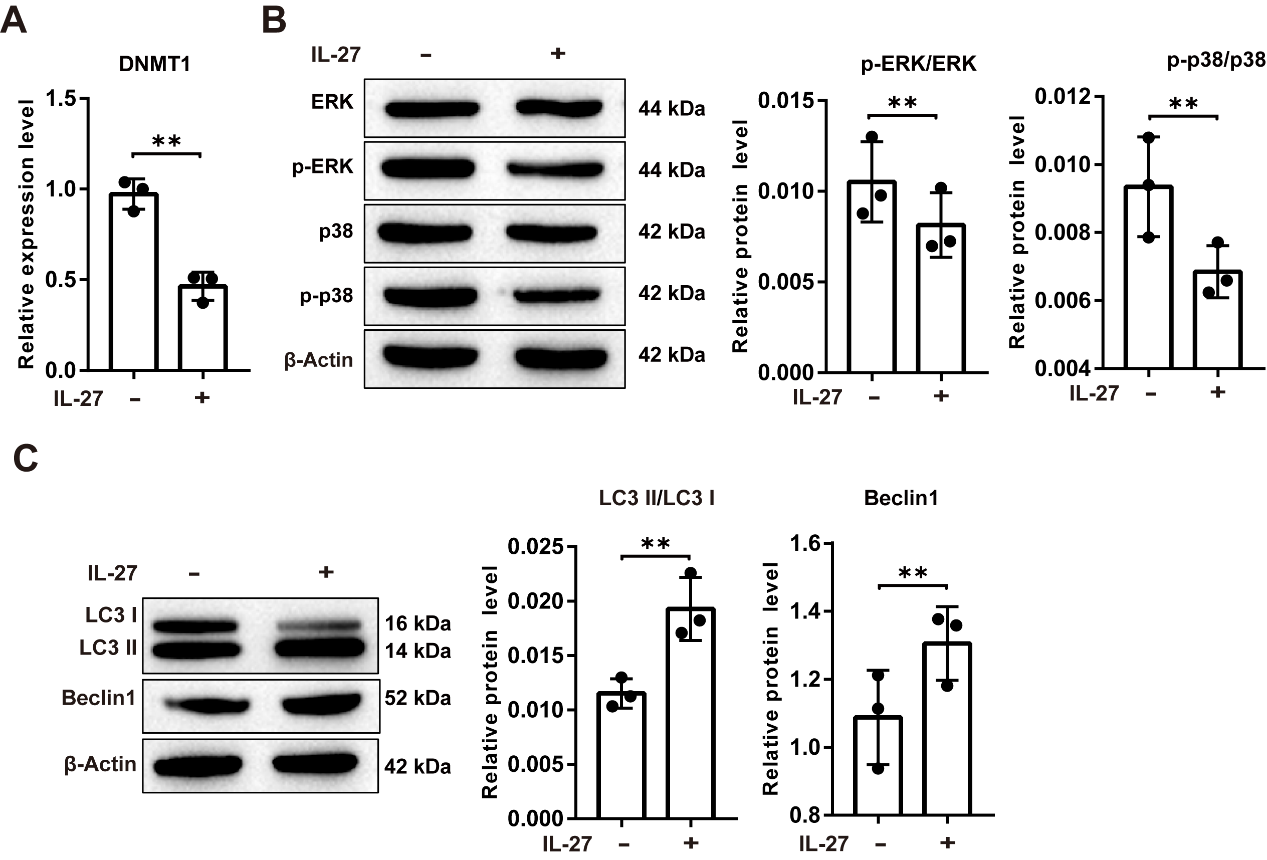


Figure S3 Effects of IL-27 on DNMT1, ERK/p38 signaling pathways and autophagy


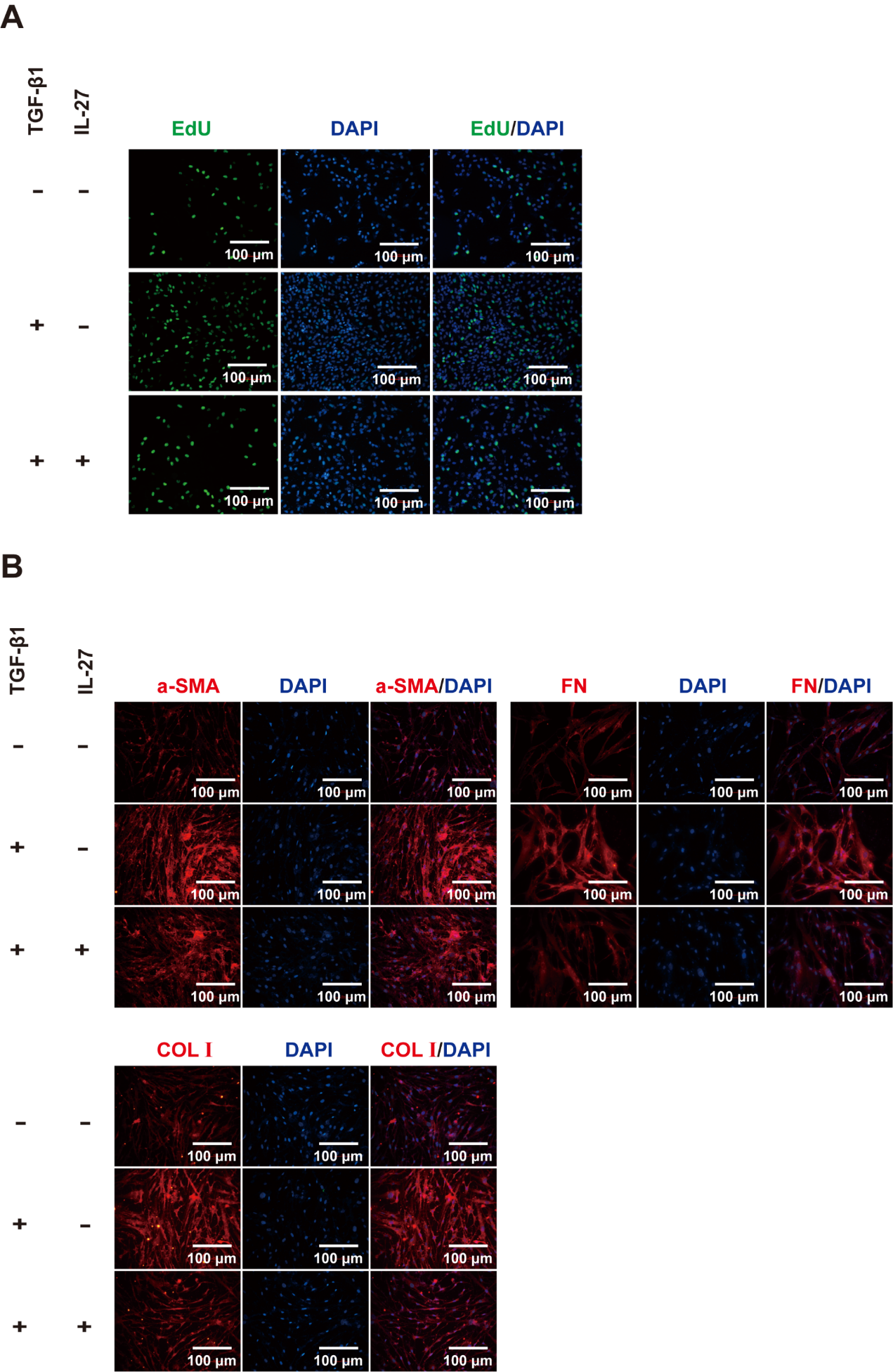


Figure S4 EdU for cell proliferation viability and immunofluorescence to detect α-SMA, FN, and COL Ⅰ fluorescence intensity


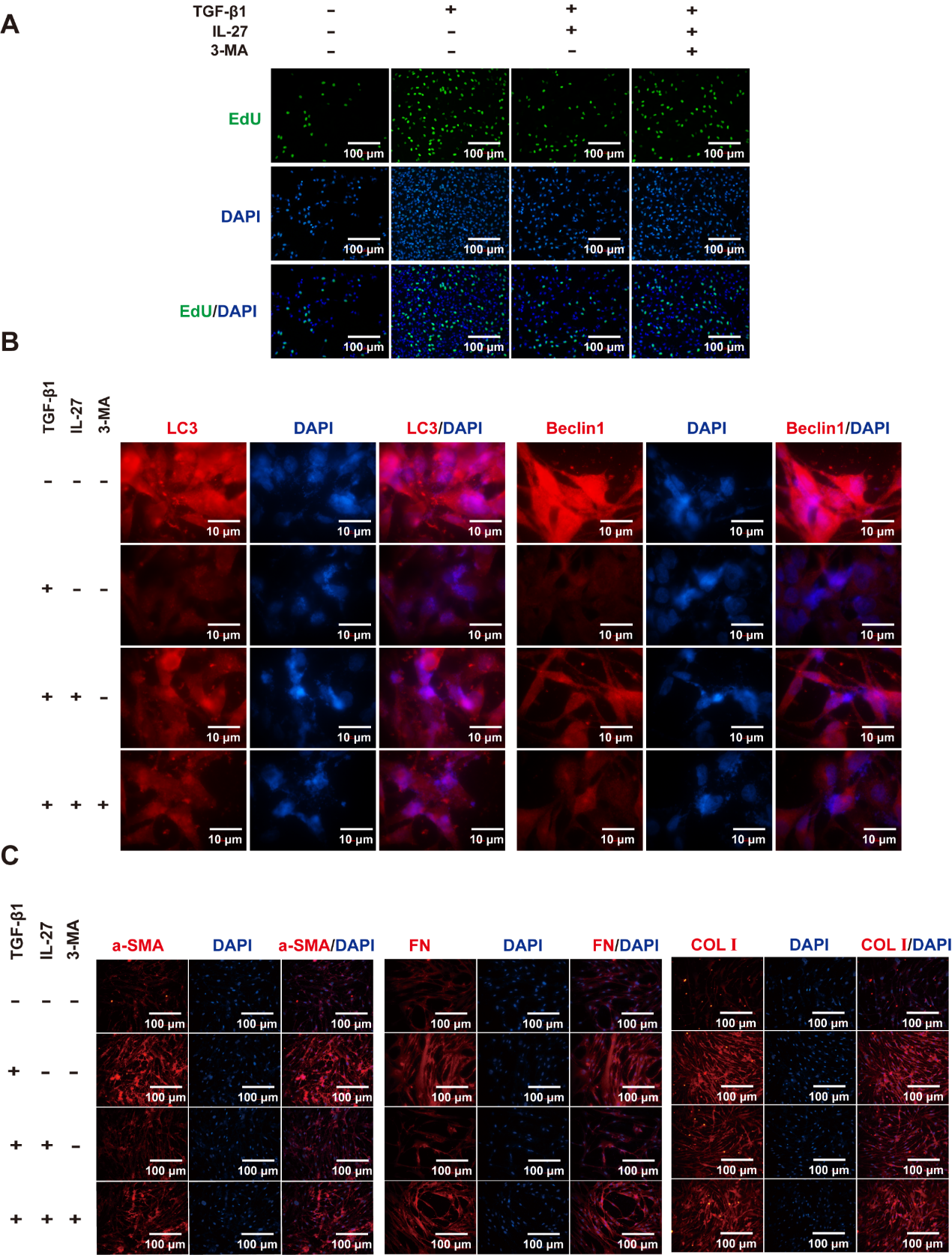


Figure S5 EdU for cell proliferation viability and immunofluorescence to detect LC3, Beclin1, α-SMA, FN, and COL Ⅰ fluorescence intensity


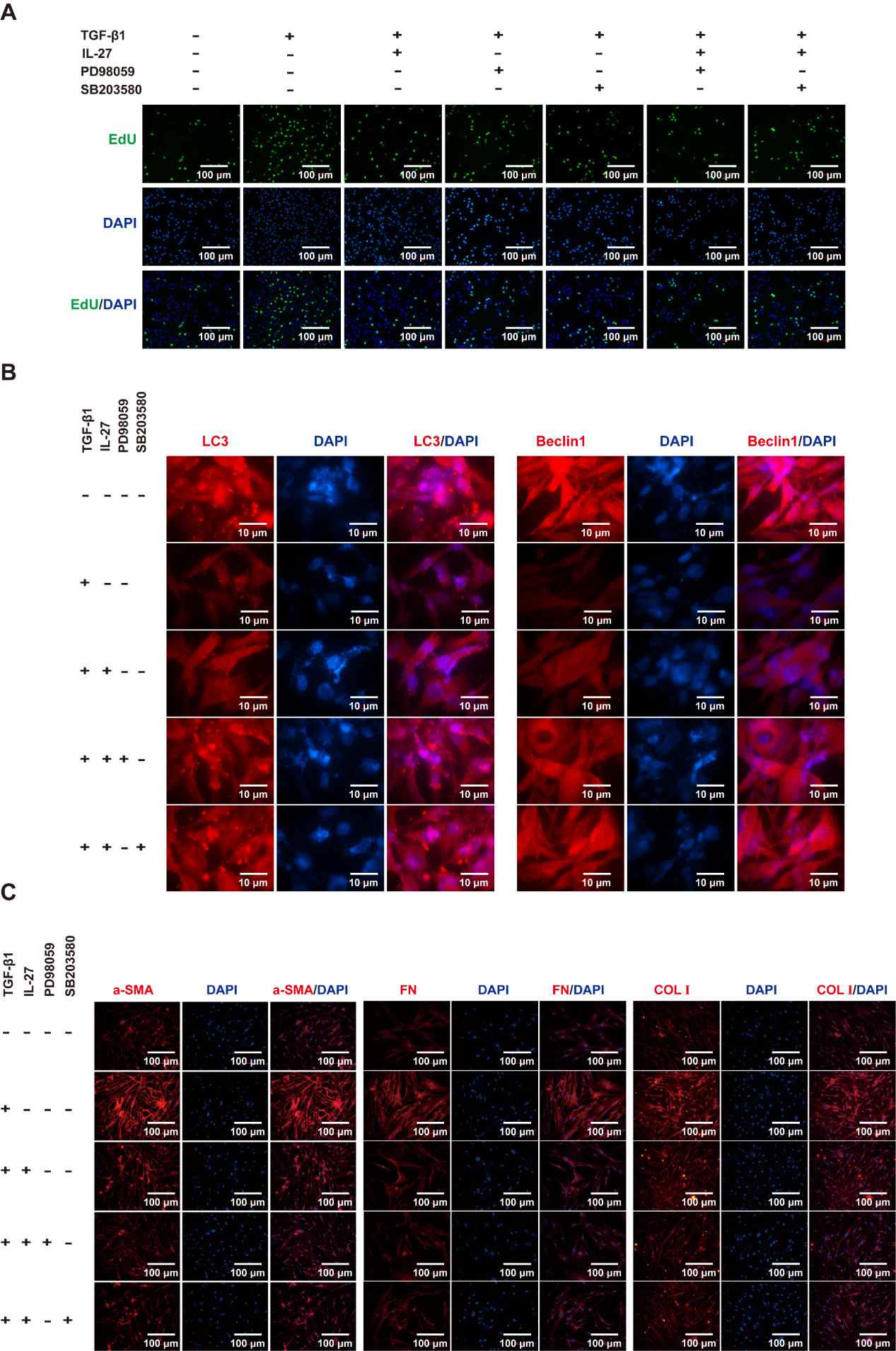


Figure S6 EdU for cell proliferation viability and immunofluorescence to detect LC3, Beclin1, α-SMA, FN, and COL Ⅰ fluorescence intensity


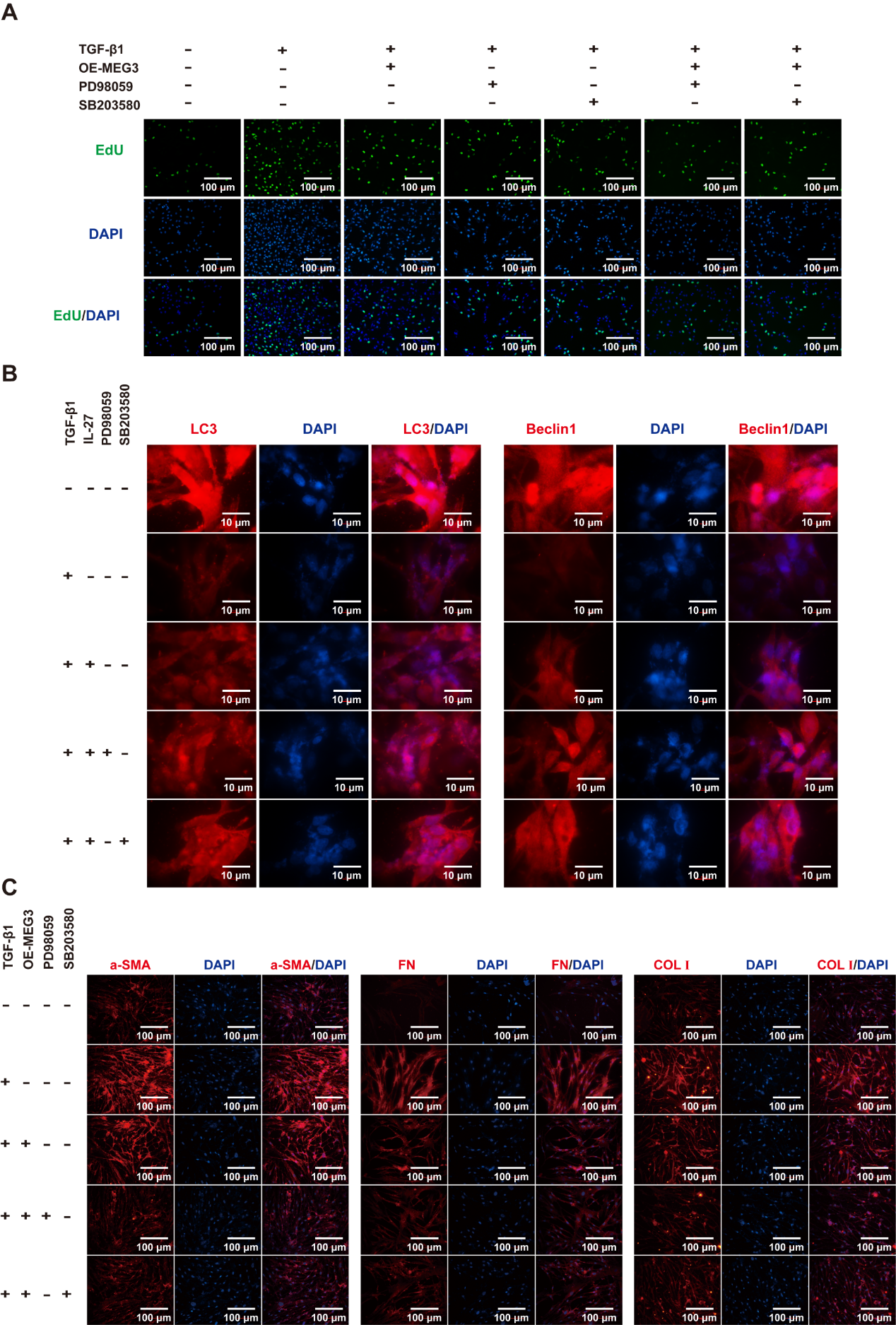


Figure S7 EdU for cell proliferation viability and immunofluorescence to detect LC3, Beclin1, α-SMA, FN, and COL Ⅰ fluorescence intensity


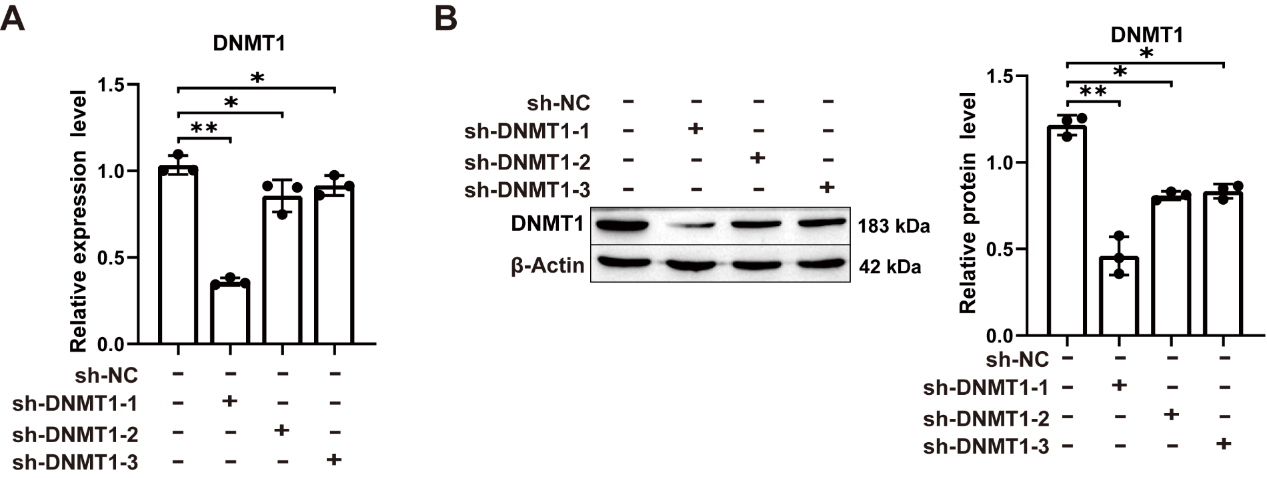


Figure S8 Detecting DNMT1 expression after sh-DNMT1 1-3 treatment by RT‒qPCR and Western blotting


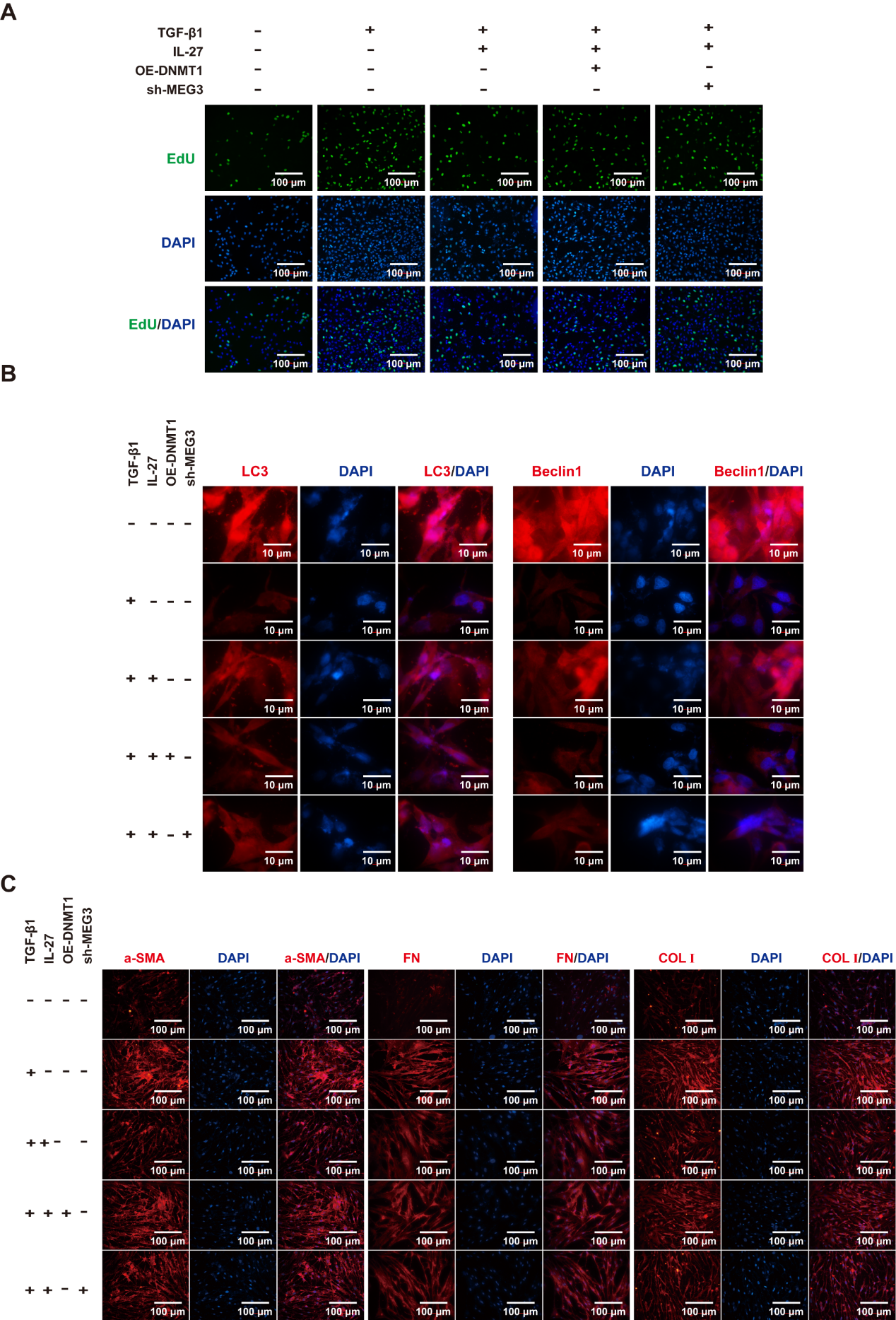


Figure S9 EdU for cell proliferation viability and immunofluorescence to detect LC3, Beclin1, α-SMA, FN, and COL Ⅰ fluorescence intensity


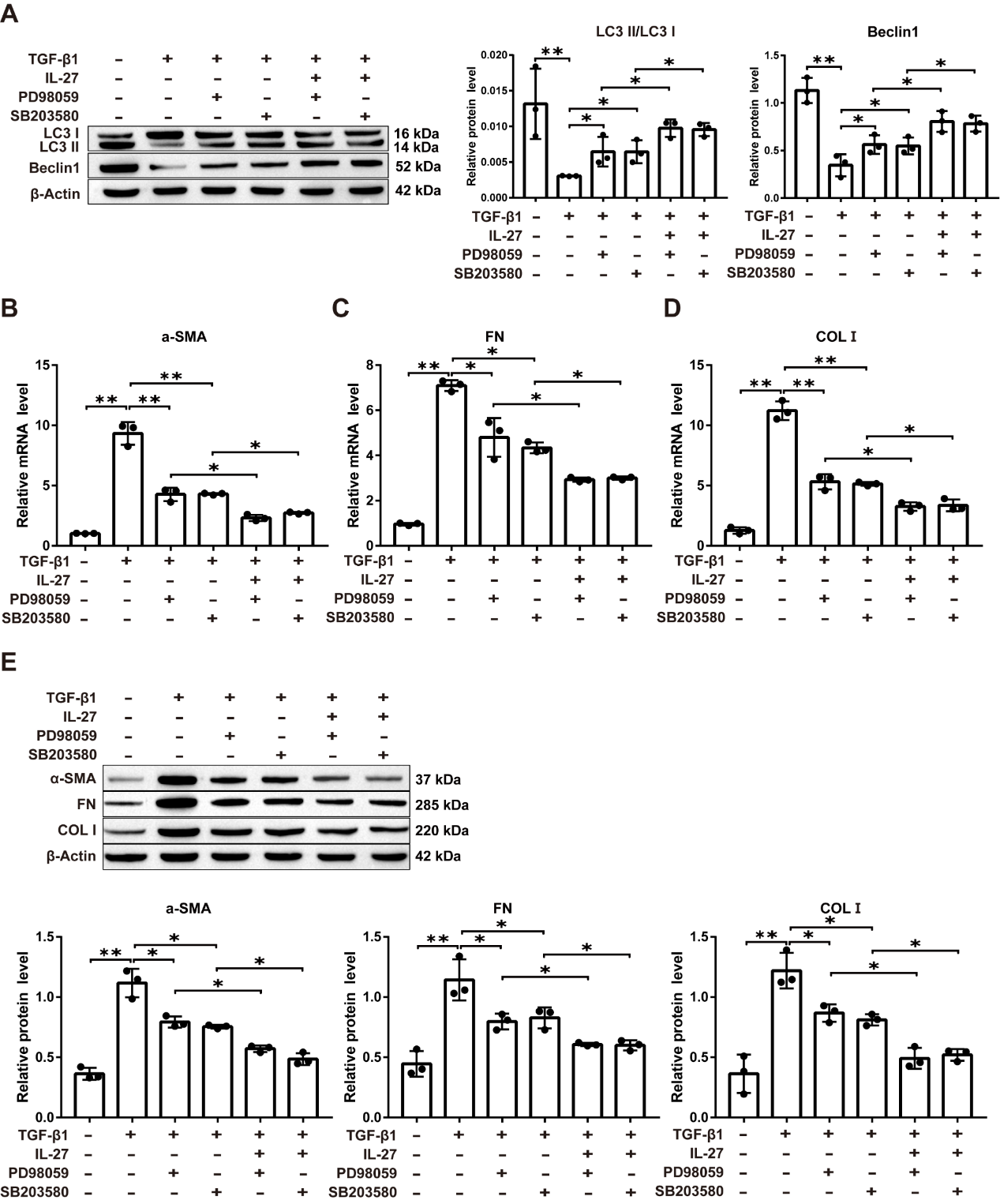


Figure S10 Detecting LC3, Beclin1, α-SMA, FN, and COL Ⅰ by RT‒qPCR and Western blotting


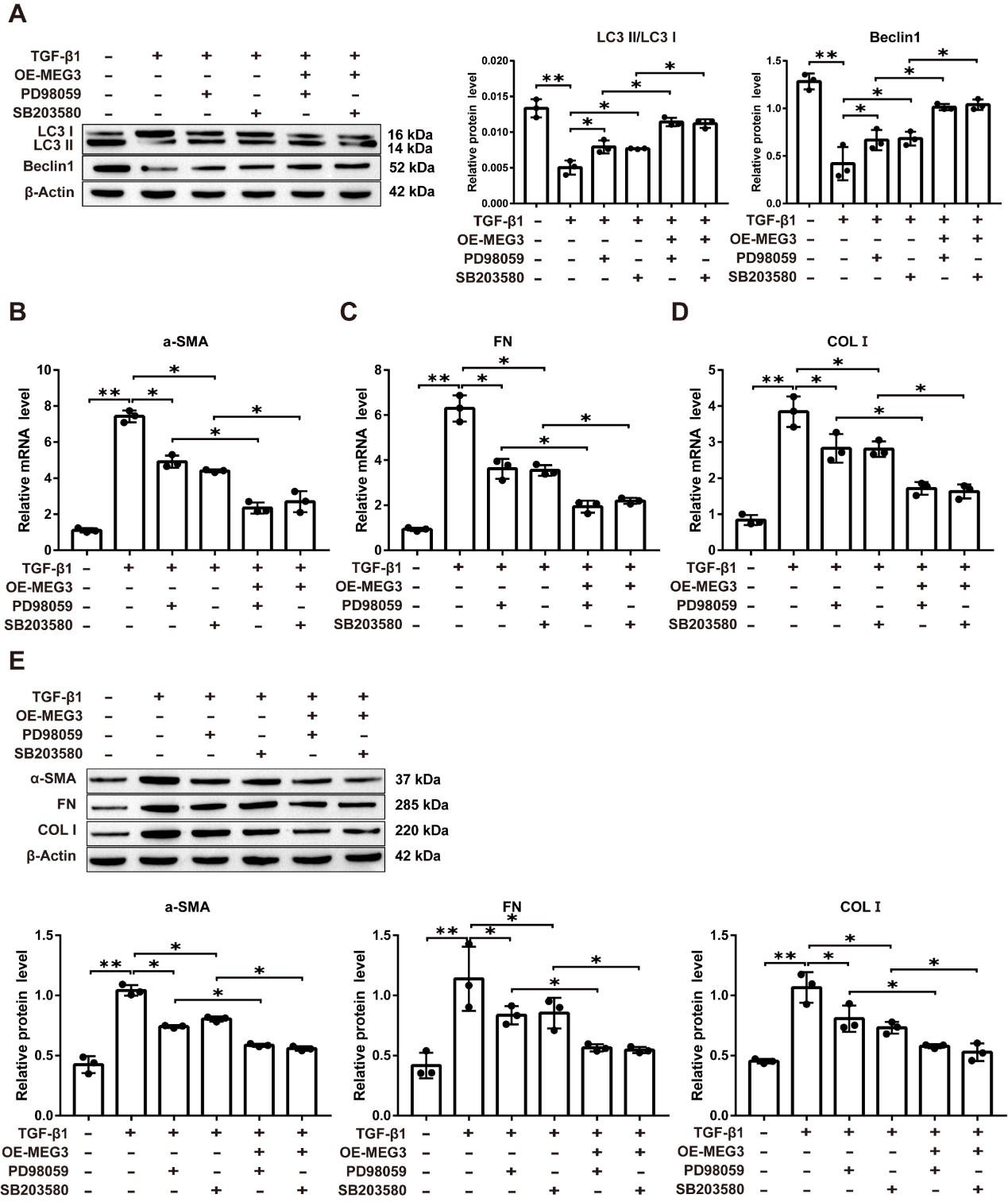


Figure S11 Detecting LC3, Beclin1, α-SMA, FN, and COL Ⅰ by RT‒qPCR and Western blotting
